# Supplementary material for: Structure-Based Virtual Screening and De Novo Design of PIM1 Inhibitors with Anticancer Activity from Natural Products
Source: Pharmaceuticals (Basel). 2021 Mar 18;14(3):275. doi: 10.3390/ph14030275 (PMC8003278; doi:10.3390/ph14030275)

Supplementary Materials

Structure-Based Virtual Screening and *De Novo* Design of PIM1 Inhibitors with Anticancer Activity from Natural Products

Hwangseo Park,[1,*] Jinwon Jeon,[2,3] Kewon Kim,[2,3] Soyeon Choi,[2,3] Sungwoo Hong[2,3,*]

[1] *Department of Bioscience and Biotechnology & Institute of Anticancer Medicine Development, Sejong University, 209 Neungdong-ro, Kwangjin-gu, Seoul 05006, Korea*

[2] *Center for Catalytic Hydrocarbon Functionalizations, Institute for Basic Science (IBS), 34141, Korea*

[3] *Department of Chemistry, Korea Advanced Institute of Science and Technology (KAIST), Daejeon 34141, Korea*

Figure S1. Spectral copies of ^{1}H- and ^{13}C- NMR data for compounds **5, 10, 13, 15,** and **16.**

(*Z*)-6-Hydroxy-2-(3-methoxybenzylidene)benzofuran-3(2*H*)-one (5)

^{1}H NMR 600 MHz, DMSO-*d₆*

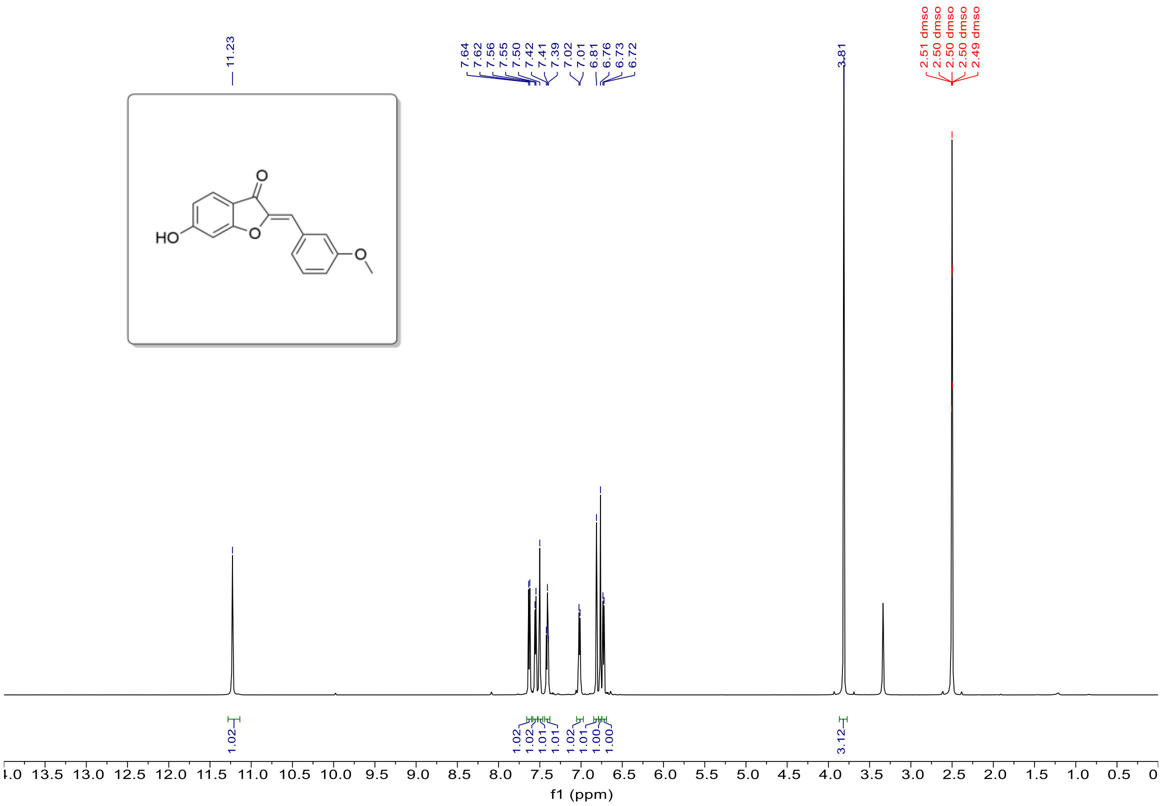

^{13}C NMR 100 MHz, DMSO-*d₆*

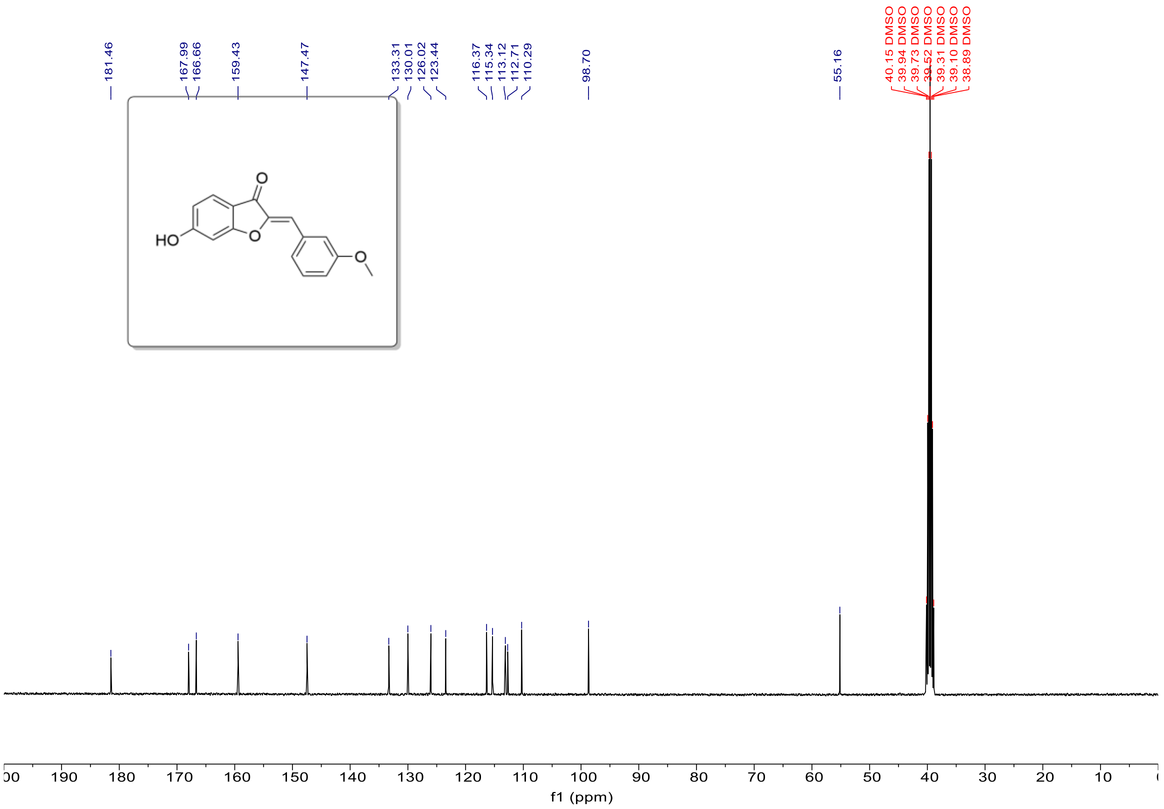

(*Z*)-2-(3,4-Dichlorobenzylidene)-4,6-dihydroxybenzofuran-3(2*H*)-one (10)

^{1}H NMR 400 MHz, DMSO-d_6

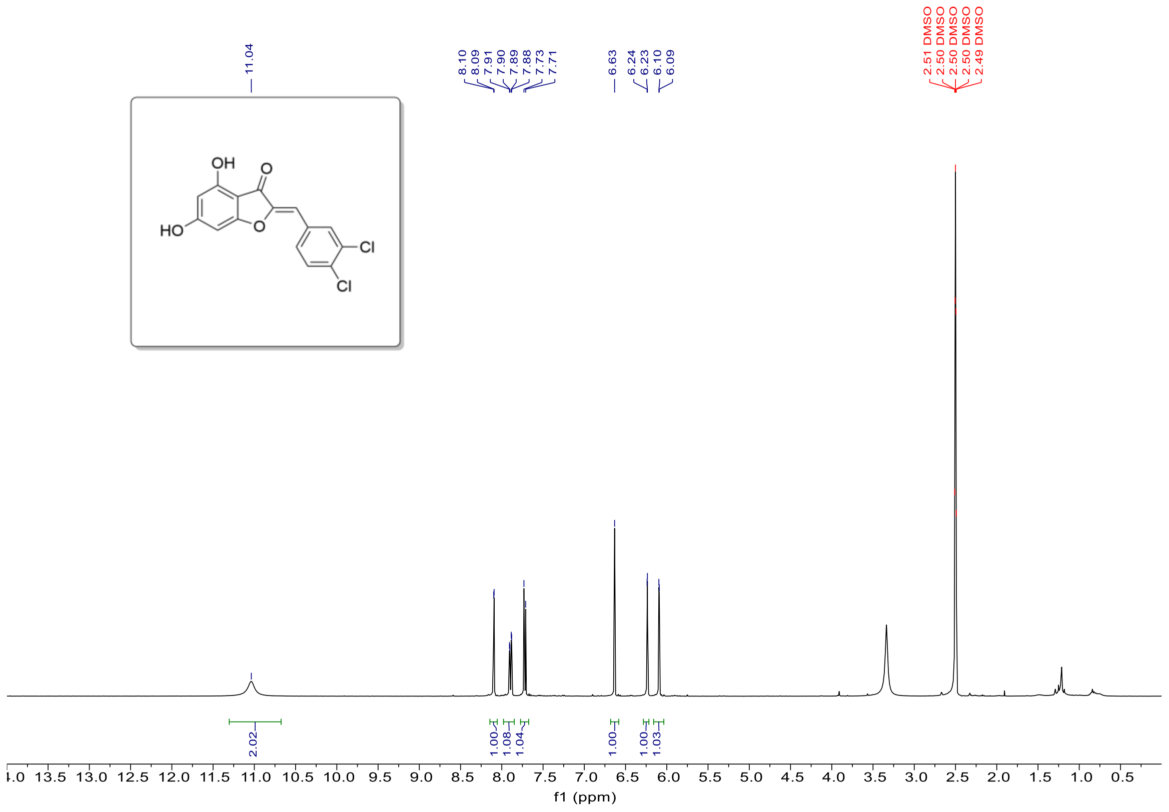

^{13}C NMR 100 MHz, DMSO-d_6

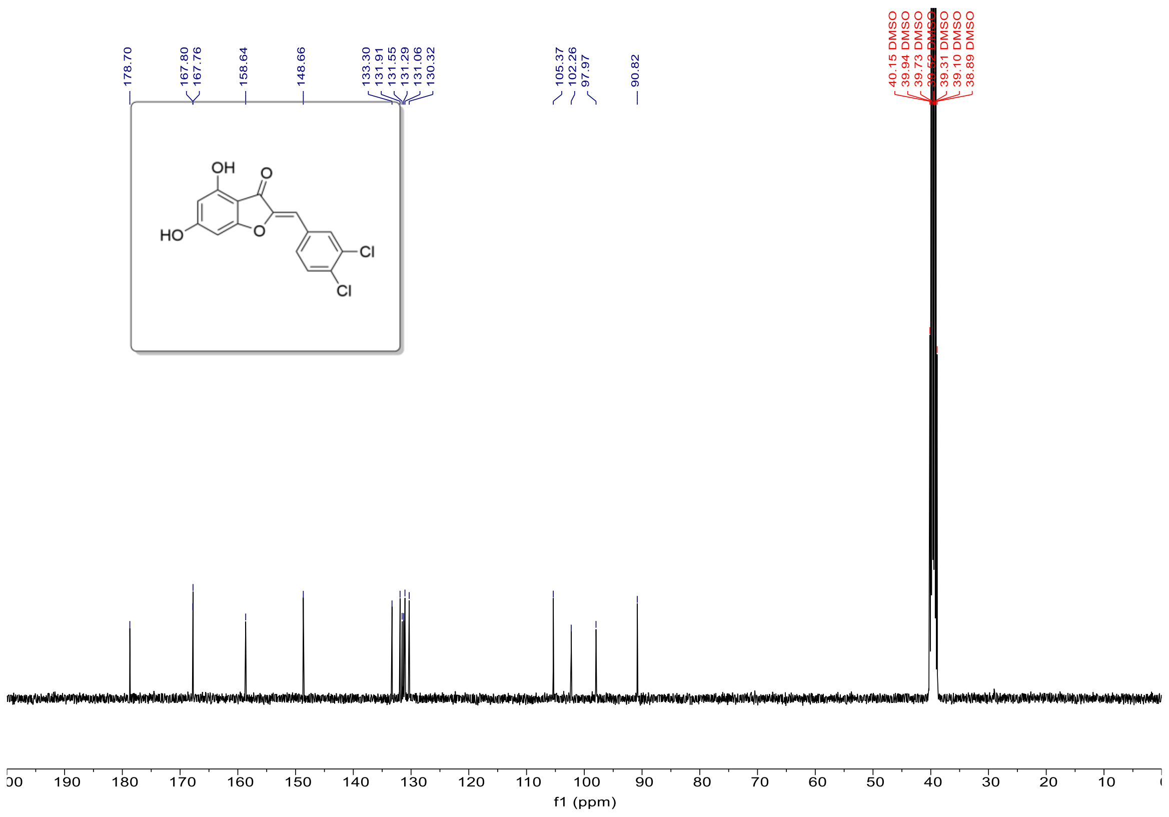

(*Z*)-3-((4,6-Dihydroxy-3-oxobenzofuran-2(3*H*)-ylidene)methyl)quinolin-2(1*H*)-one (13)

^{1}H NMR 400 MHz, DMSO-*d*$_6$

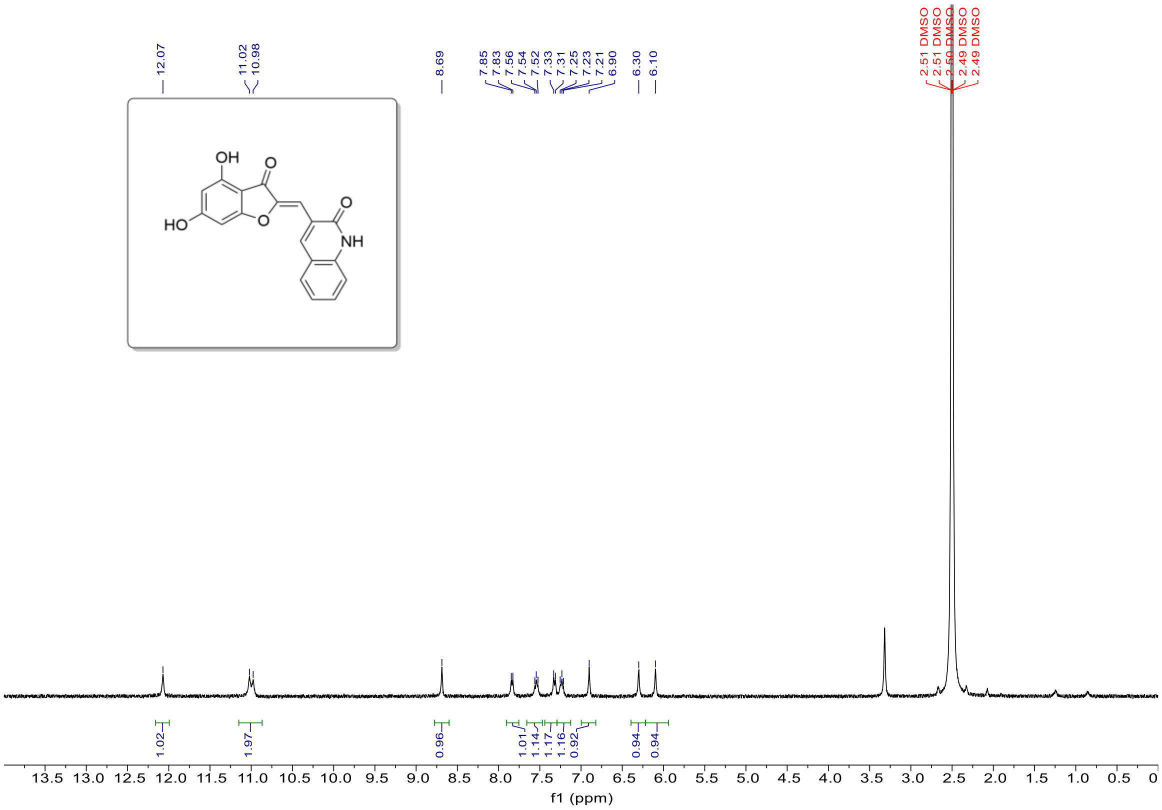

^{13}C NMR 150 MHz, DMSO-*d*$_6$

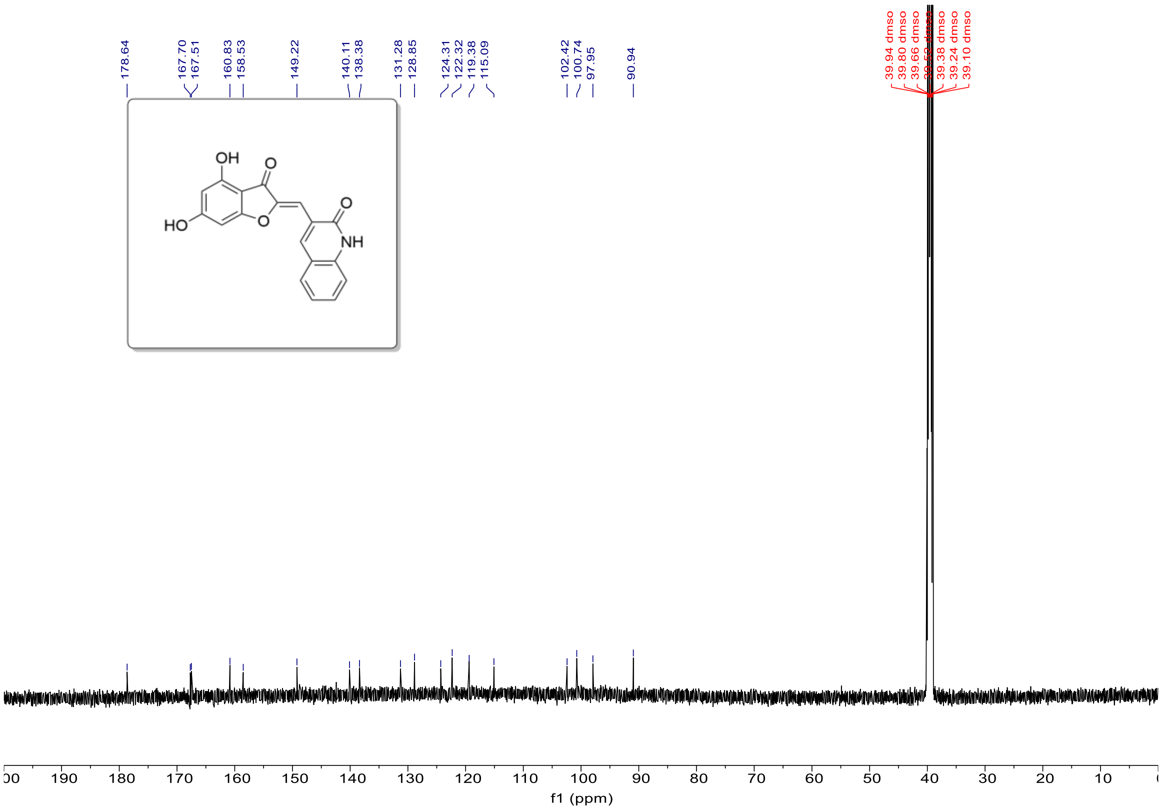

(*Z*)-2-((5-Chloro-1*H*-pyrrolo[2,3-*b*]pyridin-3-yl)methylene)-4-hydroxy-6-methoxybenzofuran-3(2*H*)-one (15)

^{1}H NMR 600 MHz, DMSO-*d*$_6$

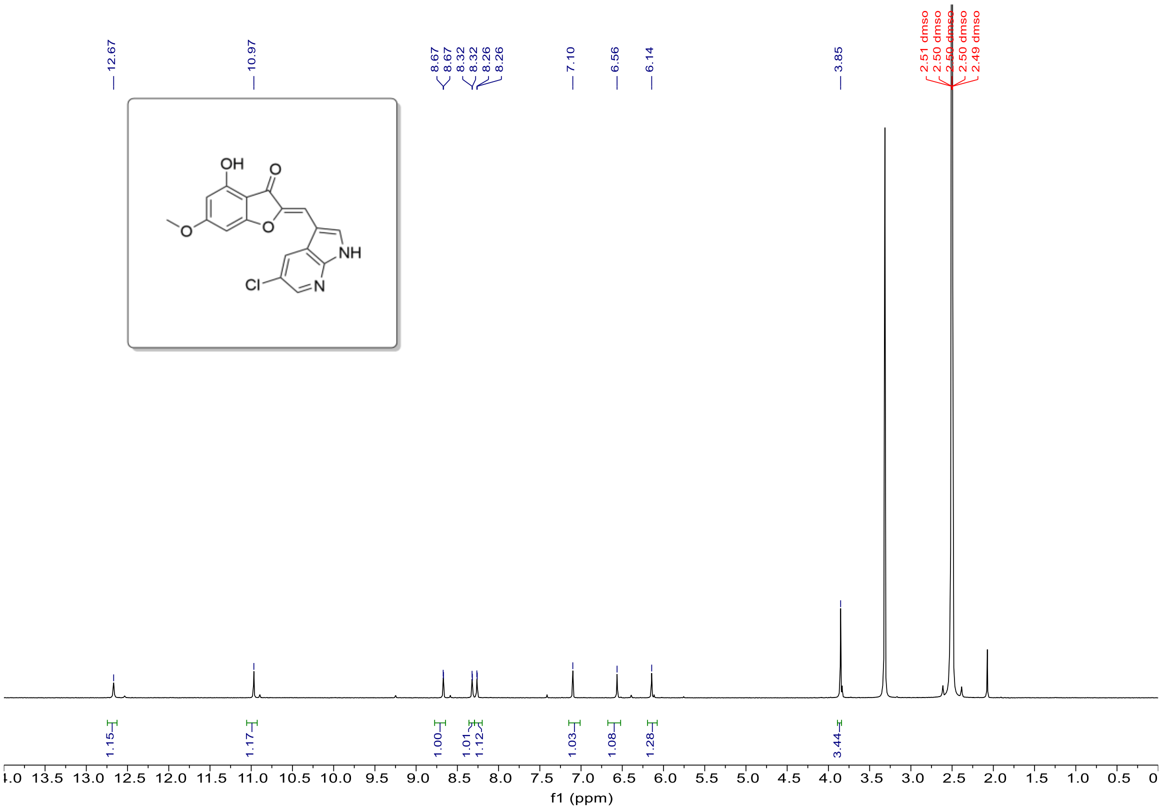

^{13}C NMR 100 MHz, DMSO-*d*$_6$

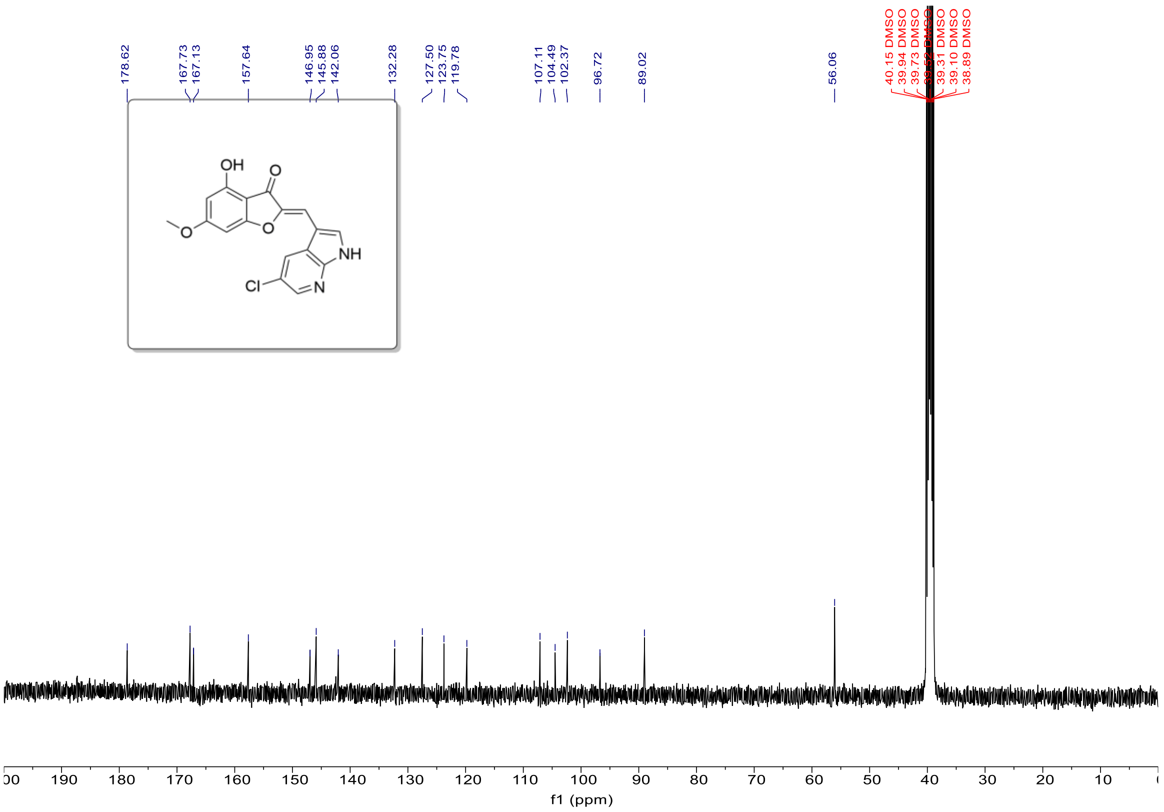

(*Z*)-2-((2-Chloro-7*H*-pyrrolo[2,3-*d*]pyrimidin-5-yl)methylene)-4,6-dihydroxybenzofuran-3(2*H*)-one (16)

^{1}H NMR 600 MHz, DMSO-*d*$_6$

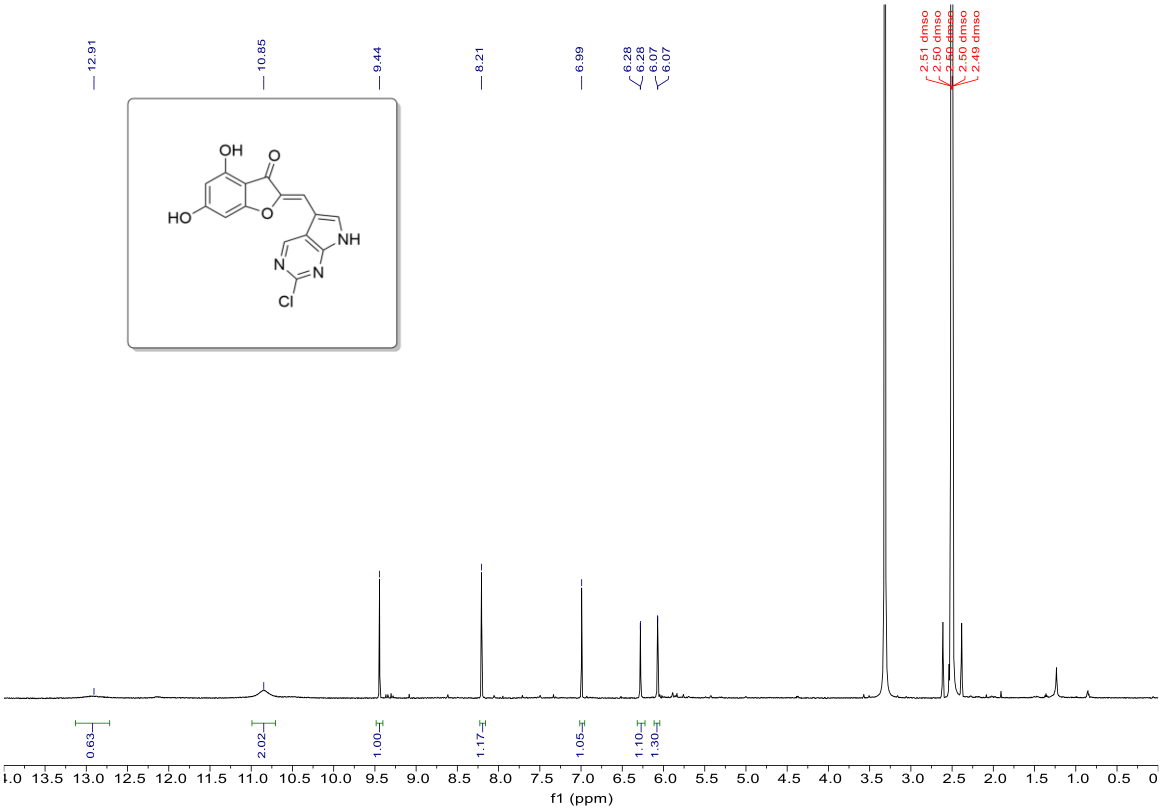

Supplement: Supplementary file 1 [file pharmaceuticals-14-00275-s001.pdf]
